# Reduced peroxisome proliferator-activated receptor-α and bile acid nuclear receptor NR1H4/FXR may affect the hepatic immune microenvironment of biliary atresia

**DOI:** 10.3389/fimmu.2022.875593

**Published:** 2022-08-25

**Authors:** Yingxuan Ma, Li Lu, Kezhe Tan, Zhi Li, Ting Guo, Yibo Wu, Wei Wu, Lulu Zheng, Feilong Fan, Jiayu Mo, Zhenhua Gong

**Affiliations:** ^1^ Department of General Surgery, Children’s Hospital of Shanghai, Shanghai Jiao Tong University, Shanghai, China; ^2^ Pathology Department, Children’s Hospital of Shanghai, Shanghai Jiao Tong University, Shanghai, China

**Keywords:** biliary atresia, bioinformatics, PPARα, NR1H4, immune microenvironment

## Abstract

**Background:**

Biliary atresia (BA) is a childhood liver disease characterized by fibrous obstruction and obstruction of the extrahepatic biliary system and is one of the most common and serious biliary disorders in infants. Significant inflammation and fibrosis of the liver and biliary tract are the most prominent features, regardless of the initial damage to the BA. Abnormalities in innate or adaptive immunity have been found in human patients and mouse models of BA. We previously reported that children with BA had abnormal lipid metabolism, including free serum carnitine.

**Objective:**

To study gene and protein expression levels of the hepatic peroxisome proliferator-activated receptor-α (PPARα) signaling pathway and farnesoid X receptor (FXR) in BA and BA fibrosis, and assess their clinical values.

**Methods:**

Low expression of PPARα and NR1H4 (FXR) in BA were validated in the Gene Expression Omnibus database. Functional differences were determined by gene set enrichment analysis based on of PPARα and NR1H4 expression. BA patients from GSE46960 were divided into two clusters by using consensus clustering according to PPARα, NR1H4, and SMAD3 expression levels, and immunoinfiltration analysis was performed. Finally, 58 cases treated in our hospital were used for experimental verification. (IHC: 10 Biliary atresia, 10 choledochal cysts; PCR: 10 Biliary atresia, 14 choledochal cysts; WB: 10 Biliary atresia, 4 choledochal cysts).

**Results:**

Bioinformatics analysis showed that the expression of PPARα, CYP7A1 and NR1H4 (FXR) in the biliary atresia group was significantly lower than in the control group. More BA-specific pathways, including TGFβ signaling pathway, P53 signaling pathway, PI3K-AKT-mTOR signaling pathway, etc., are enriched in BA patients with low PPARα and NR1H4 expression. In addition, low NR1H4 expression is abundant in inflammatory responses, IL6/STAT3 signaling pathways, early estrogen responses, IL2 STAT5 signaling pathways, and TGFβ signaling pathways. The TGFβ signaling pathway was significant in both groups. According to the expression of PPARα, NR1H4 and SMAD3, a key node in TGFβ pathway, BA patients were divided into two clusters using consensus clustering. In cluster 2, SMAD3 expression was high, and PPARα and NR1H4 expression were low. In contrast to cluster 1, immune cell infiltration was higher in cluster 2, which was confirmed by immunohistochemistry. The mRNA and protein levels of PPARα and NR1H4 in BA patients were lower than in the control group by immunohistochemistry, Western blot analysis and real-time PCR.

**Conclusions:**

The downregulation of PPARα and NR1H4 (FXR) signaling pathway may be closely related to biliary atresia.

## Introduction

Biliary atresia (BA) is an inflammatory biliary tract disease characterized by inflammation, fibrosis, and biliary tract obstruction; it is one of the most common severe biliary tract diseases diagnosed in infancy. This progressive disease has unknown etiology, but congenital or acquired immune abnormalities have been found in BA patient samples and mouse models (1, 2). Even with surgery and various immunosuppressive treatments, intrahepatic lesion progression cannot be effectively halted (3). According to Japan’s 20-year national biliary registry, which began in 1989, the self-liver survival rate is only 49%, and the surgical success rate depends on the age of the child. The 20-year overall survival rate after liver transplantation is 89%, and the overall rate of good prognosis after transplantation has improved to 97% since 2002 (4, 5). Although liver transplantation can save children’s lives, long-term immunosuppressant use and associated complications seriously affect patient quality of life. Early surgery is considered critical for postoperative jaundice relief and autohepatic survival (6).

We previously reported that pediatric BA patients have abnormal lipid metabolism such as free serum carnitine (7, 8). Carnitine is an important carrier of fatty acids; its metabolism, absorption, and utilization are regulated by the peroxisome proliferator-activated receptor alpha (PPARα) signaling pathway, and it enter cells under the action of the transporter OCTN2 (9). Impaired activation of PPARα signaling reduces carnitine utilization and bile acid synthesis and secretion, leading to activation of Nuclear Receptor Subfamily 1 Group H Member 4 (NR1H4, also known as farnesoid X receptor [FXR]) in the liver. NR1H4 (FXR) participates in hepatocyte glucose and lipid metabolism and also inhibits inflammation, fibrosis, and apoptosis (10, 11). The use of glucocorticoids, ursodeoxycholic acid, and other adjuvant therapies after Kasai surgery can increase bile secretion to improve the therapeutic effect (12). The combination of the NR1H4 (FXR) agonist obeticholic acid and a ubiquitin-like inhibitor may significantly inhibit the activation of hepatic stellate cells and inhibit liver fibrosis (11).

Innate immunity also plays a very important role in BA development and can serve as a therapeutic target (13). Cholesterol 7α-hydroxylase (CYP7A1) is the rate-limiting enzyme for bile acid formation, and bile secretion is also regulated by PPARα and FXR signaling. The PPARα pathway regulates bile acid formation and secretion *via* fibroblast growth factor 21, and it increases bile acid production by enhancing CYP7A1 activity in the classical pathway of bile acid anabolism. PPARα also upregulates the expression of multidrug-resistant protein 3 (MDR3) on the hepatocyte membrane and promotes the excretion of bile acids and bile lipids (10, 14). Under the action of carnitine palmitoyltransferase 1 (CPT1A), which is the rate-limiting enzyme for fatty acid oxidative degradation in the outer mitochondrial membrane, carnitine binds to long-chain fatty acyl-CoA to form long-chain acyl-carnitine. In BA patients, PPARα signaling pathway activation is impaired; carnitine utilization, bile acid synthesis, and secretion are reduced; and blood carnitine is increased. It is also speculated that bile synthesis and secretion are reduced, which prevents bile acids from activating FXR signaling and suppressing inflammation and fibrosis.

Here we investigated the roles of PPARα and NR1H4 (FXR) in these pathogenic processes. We analyzed BA transcriptome profiling data from the Gene Expression Omnibus (GEO) database to comprehensively understand the role of PPARα and NR1H4 (FXR) in disease immunity. Patient samples were divided into two groups according to PPARα and NR1H4 (FXR) expression, and bioinformatics techniques were used to compare differences in biological pathways. Immune cell infiltration assays and real-time polymerase chain reaction (PCR) were performed to verify the findings. Consensus clustering was used to divide BA patients in the GSE46960 dataset into two groups: cluster 1 (low expression of SMAD3, high expression of PPARα and NR1H4) and cluster 2 (high expression of SMAD3, low expression of PPARα and NR1H4). Our results indicate that patients in cluster 2 have high levels of immunologically infiltration and are better suited for immunotherapy.

## Materials and methods

### Data acquisition and organization

The BA transcriptome dataset GSE46960 was obtained from the public Gene Expression Omnibus (GEO) database; it included liver tissue from 64 cases of BA, 14 non-BA patients with intrahepatic cholestasis, and 7 normal samples (control). The differentially expressed genes of BA liver tissues (64 cases) and control group liver tissues (21 cases) were compared and analyzed with online GEO2R software. Histograms for the two groups were drawn according to the expression levels of PPARα, OCTN2, CPT1A, CYP7A1, MDR3, and NR1H4 (FXR). Differences were considered significant at P<0.05.

### Gene enrichment analysis

According to previous studies, free carnitine in BA patients can be used to diagnose BA, while PPARα and NR1H4 (FXR) are key genes in the PPAR signaling pathway and bile acid metabolism (7) with significantly reduced expression in BA. Gene set (h.all.v7.4.symbols.gmt) was used for gene set enrichment analysis (GSEA), which is a computational method that determines whether an *a priori* defined set of genes shows statistically significant, concordant differences between two biological states (15), which was performed for PPARα and NR1H4 (FXR) expression. Potentially involved signaling pathways were evaluated, and their molecular mechanisms in BA development were investigated.

Gene expression was divided into high and low groups according to median PPARα and NR1H4 (FXR) expression levels, and 1000 times set sequences were performed for each analysis. The expression levels of PPARα and NR1H4 (FXR) are given as the normalized enrichment score (NES), normalized significance level (nominal P value), and adjusted multiple hypothesis tests (false discovery rate [FDR] q value). Pair enrichment pathways for each phenotype are classified. Gene sets with NES≥1.0, nominal P value ≤ 0.05, and FDRq value ≤ 0.25 were identified as meaningful.

### Protein-protein interaction (PPI) networks and screening for key *genes in signaling pathways*

PPI networks in meaningful signaling pathways were obtained through the online analysis website STRING (https://string-db.org/) and exported in.TSV format. The obtained source file was imported into the open source software platform Cytoscape (https://cytoscape.org/) for visual analysis, and the plug-in cytoHubba was used for hub gene analysis to screen for the key gene SMAD family member 3 (SMAD3).

### Consensus clustering and immune cell infiltration analysis based *on SMAD3, PPARα, and NR1H4*

Extraction and clustering of SMAD3, PPARα, and NR1H4 expression was performed with the R package Consensus Cluster Plus (16). The sample was divided into two clusters.

CIBERSORT is a method of calculating cell composition based on an expression profile and can be used for almost any tissue. This algorithm was used to calculate the proportions of 22 immune cells in each BA patient. The sum of the 22 immune cell group fractions for each sample is 1 (17).

The degrees of infiltration of 28 immune cells were calculated based on the levels of gene expression in the 28 published immune cell genomes by applying the R Package GSVA Single Sample GSEA (ssGSEA) method. Data set GSE15235 is used for external validation.

### Human samples

This study was reviewed and approved by the Institutional Review

Board of the Shanghai Children’s Hospital. A total of 30 of BA、28 of choledochus cyst (CC) patients’ liver were used for verification. Liver samples of 10 BA and 10 CC patients were prepared as paraffin sections for immunohistochemistry, the remaining 38 were frozen and stored in liquid nitrogen for real-time PCR and western blot analyses.

### Immunohistochemistry

Six paraffin sections approximately 4-μm thick were excised from each tissue block and used for immunohistochemical labeling with six antibodies purchased from Abcam (Cambridge, UK): recombinant anti-PPAR alpha [EPR21244], anti-solute carrier family 22 member 5 antibody, anti-CPT1A antibody, anti-CYP7A1 antibody, anti-ABCB4 antibody targeting the N-terminal, and human NR1H4 (FXR)/NR1H4 antibody.

Immunohistochemical staining was performed for PPARα, NR1H4 (FXR), OCTN2, CPT1A, CYP7A1, and MDR3 (ABCB4) using the above-mentioned antibodies. Since PPARα and NR1H4 (FXR) are expressed in the nucleus, immunohistochemical staining shows positive expression in the nuclei of hepatocytes. OCTN and MDR3 (ABCB4) were expressed on the cell membrane, and brownish-yellow staining of the hepatic cell membrane was considered as positive expression. Since CPT1A is expressed in the outer mitochondrial membrane and CYP7A1 is expressed in the endoplasmic reticulum and mitochondria, immunohistochemical staining revealed a positive brownish yellow color in the cytoplasm of the liver. All samples were processed with positive and negative controls.

### Real-time PCR detection of NR1H4 (FXR), PPARα, and SMAD3

Real-time PCR was performed to detect the expression of NR1H4 (FXR), PPARα, and SMAD3 in liver tissues from both groups of patients. Ten frozen BA specimens were used as the experimental group, and fourteen with choledochal cysts were used as the control group. Liver tissue was minced and mixed with 0.5 ml of TRIzol to completely dissolve the samples, which were centrifuged at 12,000 *g* for 5 min at 4°C. The supernatant was collected and added to 250 μl of chloroform, total RNA was extracted by the conventional TRIzol method, and the absorbance was measured in order to calculate the amounts of 1-μg RNA samples. We prepared a well-mixed 20 μl reverse transcription reaction system that was placed in a PCR machine for reverse transcription. We prepared a 10-μl real-time fluorescent quantitative PCR reaction system. The primer sequences for the target genes and the control glyceraldehyde-3-phosphate dehydrogenase (*GAPDH*) gene are listed in **Table 1**. Relative expression of the target gene was calculated by the 2-ΔΔCt method. Th amplification conditions were 95°C for 20 min, 95°C for 30 s, 58°C for 30 s, 40 cycles, 72°C for 40s.

**Table 1:** RT-PCR primers.

**NR1H4-173-F**	**5’-TTTTGACGGAAATGGCAACC-3’**
**NR1H4-173-R**	5’-CCCAGACGGAAGTTTCTTATTGA-3’
**PPARa-147-F**	5’- ATCAAGTGACATTGCTAAAATACGG-3’
**PPARa-147-R**	5’-CACAGAACGGTTTCCTTAGGCT-3’
**SMAD3-157-F**	5’-GGAGCGGAGTACAGGAGACAGAC-3’
**SMAD3-157-R**	5’-CTAAGACACACTGGAACAGCGGATG-3’
**Human GAPDH-F**	5’- GCACCGTCAAGGCTGAGAAC -3’
**Human GAPDH-R**	5’- TGGTGAAGACGCCAGTGGA -3’

F, forward; R, reverse; GAPDH was used as an internal control.

### Western blot detection of NR1H4 (FXR), PPARα, and SMAD3

Western blotting was performed to detect the expression of NR1H4 (FXR), PPARα, and SMAD3 in liver tissue. Approximately 100 mg of frozen tissue was thawed in 1 ml of protein lysis buffer, and total protein was extracted by an ultrasonic method. Protein concentrations were determined using the bicinchoninic acid method. Equal samples were loaded for gel electrophoresis, the proteins were then transferred to membranes, which were blocked prior to adding the primary antibody for overnight incubation overnight at 4°C. The next day, the membranes were incubated with an appropriate secondary antibody for 1 hour at room temperature. Finally, the blot was incubated in a coloring solution, and the signal was developed after rinsing.

### Statistical analysis

GraphPad Prism 9 (GraphPad Software, Inc, San Diego, CA, USA) was used to generate graphs, and SPSS 26.0 software (IBM Corp, Armonk, NY, USA) was used for data processing, measurement data (mean ± standard deviation), and immunohistochemical semi-quantitative analysis in the BA group and common bile duct cyst group. The integrated optical density levels of the two groups were non-normally distributed continuous measurement data. Differences were compared using rank-sum tests, and P<0.05 was considered statistically significant.

## Results

### Analysis of differentially expressed genes

**Figure 1:**
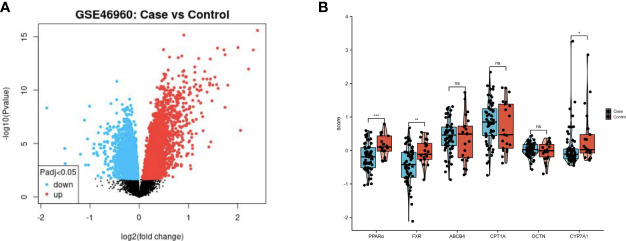
PPAR and FXR were downregulated in the liver of patient with BA. **(A)** Volcano plot of the GSE46960 dataset (BA patients: n=64, non-BA patients with intrahepatic cholestasis: n=14, and normal liver patients: n=7). Red and blue points indicate up- and downregulated genes in BA group respectively. **(B)** Comparison of gene expression levels between two clusters. ns, not significant, *P<0.05, **P<0.01, ***P<0.001.

The GSE46960 dataset was downloaded from the GEO database and processed with GEO2R software. It included samples of between BA liver tissue (64 patients) and control liver tissue (14 non-BA patients with intrahepatic cholestasis, 7 normal liver patients). Gene expression levels were analyzed, and genes with adjusted P-values (Padj)<0.05 were considered as differentially expressed (**Figure 1A**). Histograms were drawn according to the expression levels of PPARα, OCTN2, CPT1A, CYP7A1, MDR3, and NR1H4 (FXR). PPARα, CYP7A1 and NR1H4 (FXR) were significantly differentially expressed between the two groups (**Figure 1B**).

### Gene enrichment analysis

**Figure 2:**
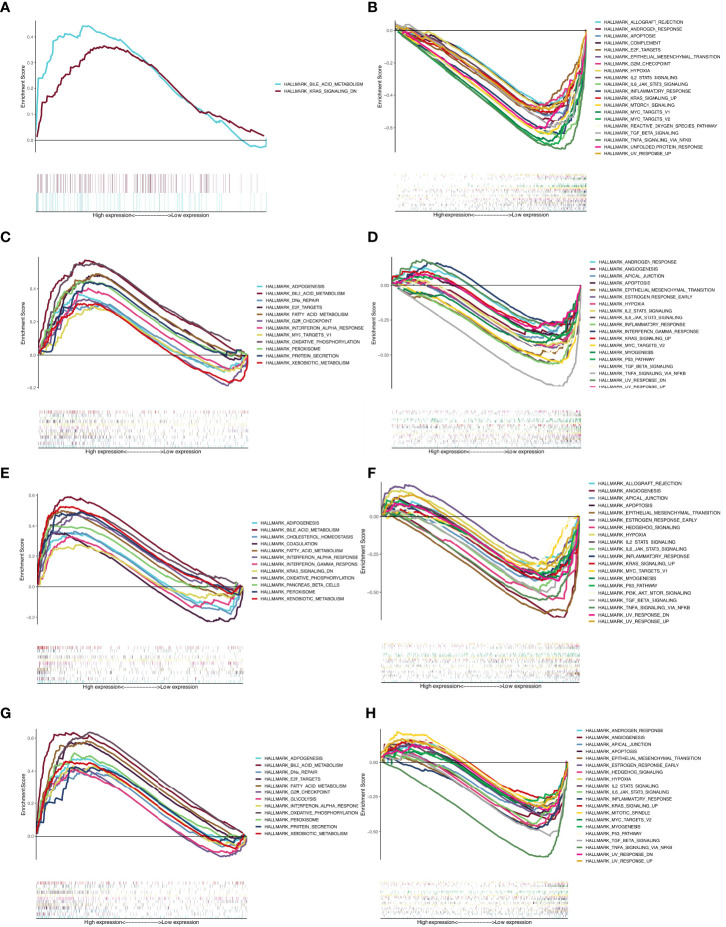
GSEA results for the BA group. **(A)** The enriched pathway in PPARα high expression group in GSE46960; **(B)** The enriched pathway in PPARα low expression group in GSE46960; **(C)** The enriched pathway in NR1H4 high expression group in GSE46960; **(D)** The enriched pathway in NR1H4 low expression group in GSE46960; **(E)** The enriched pathway in PPARα high expression group in GSE15235; **(F)** The enriched pathway in PPARα low expression group in GSE15235; **(G)** The enriched pathway in NR1H4 high expression group in GSE15235; **(H)** The enriched pathway in NR1H4 low expression group in GSE15235.

The gene set h.all.v7.4.symbols.gmt was used for GSEA to assess response pathways that may be involved in PPARα and NR1H4 (FXR) signaling and BA development and progression. Gene expression data were divided into high and low expression groups according to PPARα and NR1H4 (FXR) levels in GSE46960, and the results were validated in 47 BA patients in the GSE15235 dataset. Results that were not statistically significant in both datasets were excluded, and the same proportions of results from both groups were retained. For PPARα and NR1H4 expression we used liver biopsy dataset GSE65359 (84 cases of hepatitis B), fetal and adult liver sample dataset GSE61276 (106 cases), and cholestasis gene expression excluding BA. Data were also selected from the GSE46960 (14 cases) and the GSE112790 liver cancer dataset (183 cases). The results showed that more BA-specific pathways were enriched in BA patients with low PPARα and NR1H4 expression. When PPARα expression is low, signaling is enhanced in the transforming growth factor-β (TGFβ), p53, and phosphoinositide 3-kinase (PI3K)-AKT-mammalian target of rapamycin (mTOR) pathways (**Figures 2A, B, E, F**). Low NR1H4 expression is associated with inflammatory responses, interleukin (IL)6/STAT3 signaling, early estrogen response, IL2 STAT5 signaling, TGFβ signaling, apoptosis, and angiogenesis (**Figures 2C, D, G, H**). TGFβ signaling was significant in both groups. We found that the same signaling pathway was not screened in the PPAR and FXR high expression groups. The TGFβ signaling pathway in the low expression group was significant in both groups (**Table 2**).

**Table 2:** GSEA enrichment analysis results for high and low expression of PPARα and NR1H4 in BA patients, and GSEA results excluding patients with hepatitis B, normal liver, liver cancer, and non-BA cholestasis.

Gene	Expression	GSEA	NES	NOM p-val	FDR q-val	FWER p-val
*PPARα*	PPARα High Expression Group	HALLMARK KRAS SIGNALING DN	1.85	0	0	0.01
	PPARα Low Expression Group	HALLMARK TGF BETA SIGNALING	-2.28	0	0	0
		HALLMARK P53 PATHWAY	-2.05	0	0	0
		HALLMARK UV RESPONSE DN	-1.87	0	0	0.01
		HALLMARK PI3K AKT MTOR SIGNALING	-1.84	0	0	0.01
*NR1H4*	NR1H4 High Expression Group	HALLMARK DNA REPAIR	1.38	0.02	0.05	0.59
	NR1H4 Low Expression Group	HALLMARK INFLAMMATORY RESPONSE	-2.79	0	0	0
		HALLMARK IL6 JAK STAT3 SIGNALING	-2.44	0	0	0
		HALLMARK ESTROGEN RESPONSE EARLY	-2.23	0	0	0
		HALLMARK IL2 STAT5 SIGNALING	-2.17	0	0	0
		HALLMARK TGF BETA SIGNALING	-2.03	0	0	0
		HALLMARK APOPTOSIS	-1.77	0	0	0.01
		HALLMARK ANGIOGENESIS	-1.59	0.01	0.01	0.06
		HALLMARK KRAS SIGNALING UP	-1.55	0	0.01	0.08
		HALLMARK ANDROGEN RESPONSE	-1.55	0	0.01	0.09
		HALLMARK UV RESPONSE UP	-1.54	0	0.01	0.1
		HALLMARK UV RESPONSE DN	-1.44	0.01	0.02	0.21

### PPI networks and screening for key genes in signaling pathways

**Figure 3:**
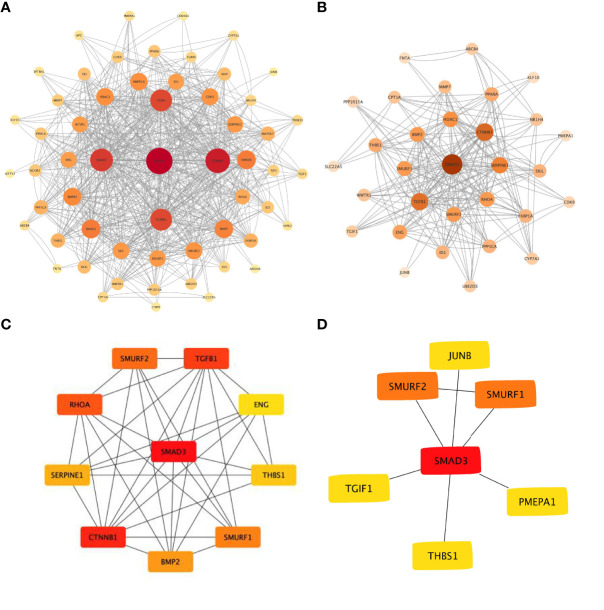
Protein-protein interaction network of TGFβ pathway **(A)** TGFβ pathway and PPARα, NR1H4, and other proteins. **(B)** GSEA of the TGFβ pathway and the interaction with PPARα, NR1H4, and other proteins based on the CORE ENRICHMENT results. **(C)** Hub gene screening with cytoHubba. **(D)** Common key genes in TGFβ pathway.

The online analysis website STRING was used to generate a network of interactions between TGFβ pathway proteins and PPARα, NR1H4, and other proteins. The resulting PPI network had a total of 57 nodes, 345 edges, and PPI enrichment P<1.0e-16. Visual analysis was performed with Cytoscape (**Figures 3A, B**), and cytoHubba was used for hub gene analysis. The potential major hub gene was identified as SMAD3 (**Figure 3C** and **Table 3**). We then screened all genes contributing to TGFβ signaling in these two data sets of biliary atresia and confirmed that SMAD3 played an important role in both data sets (**Figure 3D**). We speculated that SMAD3 activated the TGFβ pathway by interacting with PPARα and NR1H4, thereby promoting BA-related biliary fibrosis.

**Table 3:** Top 10 STRING interactions of genes screened by the MCC method.

Rank	Name	Score
**1**	SMAD3	1543
**2**	CTNNB1	1376
**3**	TGFB1	1267
**4**	RHOA	984
**5**	SMURF2	894
**6**	SMURF1	882
**7**	BMP2	804
**8**	SERPINE1	576
**9**	THBS1	384
**10**	ENG	300

### Immune cell infiltration analysis

**Figure 4:**
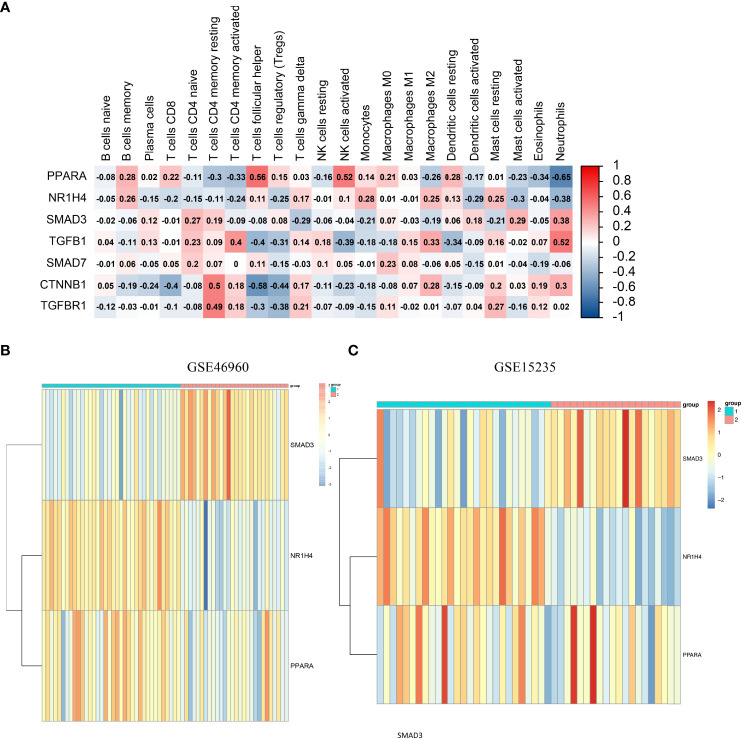
Consensus clustering and immune cell infiltration analysis based on SMAD3, PPARα, and NR1H4. **(A)** Correlation heatmap of PPARα, NR1H4, and the major genes of the TGFβ pathway. **(B)** GSE46960 patient rooting PPARα, NR1H4 (FXR), and SMAD3 divisions. **(C)** GSE15235 patient rooting PPARα, NR1H4 (FXR), and SMAD3 divisions.

A correlation heat map was created for PPARα and NR1H4, which are the major genes of the TGFβ pathway (**Figure 4A**). Considering the important role of SMAD3 in the TGFβ pathway and its close relationship with PPARα and NR1H4, we included PPARα, NR1H4 (FXR), and SMAD3 in the follow-up analysis. We performed consistent clustering of 64 BA samples based on PPARα, NR1H4 (FXR), and SMAD3 expression matrices and split the samples into two clusters by median. The heat map showed low expression of SMAD3 and high expressions of PPARα and NR1H4 (FXR) in cluster 1 (n=36), and high expression of SMAD3 and low expression of PPARα and NR1H4 (FXR) in cluster 2 (n=28) (**Figure 4B**). Next we investigated the Spearman correlations between the three genes separately. We observed a positive correlation between PPARα and NR1H4 (R=0.48, P<0.001), and negative correlation between SMAD3 and PPARα (R=-0.41, P<0.001), as well as SMAD3 and NR1H4 (R=-0.67, P<0.001). Consistent clustering was also carried out for the samples in the validation group GSE15235 according to the three genes, and similar heat maps were obtained. The 47 samples were divided into groups 1 cluster 1 (n=27) and cluster 2 (n=20) (**Figure 4C**).

### Immune cell infiltration comparison

**Figure 5:**
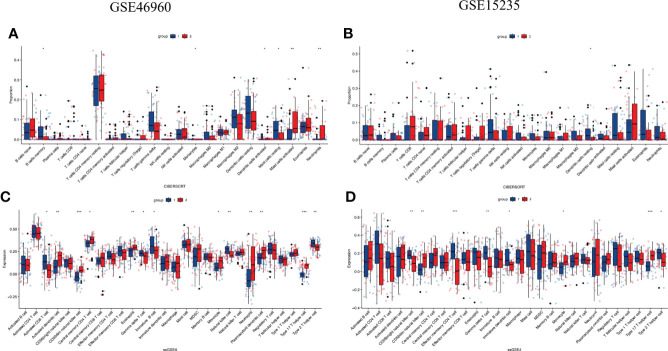
Comparison of immune characteristics between two clusters and verified by GSE15235 **(A, B)** Proportion of immune cells and **(C, D)** expression of immune cells between two clusters. The P values are labeled using asterisks *P < 0.05, **P < 0.01, ***P < 0.001.

We performed CIBERSORT analysis and ssGSEA to better understand differences in immune function. CIBERSORT analysis showed that cluster 2 had high proportions of activated mast cells (MCs) and neutrophils (**Figure 5A**). These were not confirmed in the validation set (**Figure 5B**).

ssGSEA showed 28 immune cell subtypes. The results revealed that activated dendritic cells, CD56dim natural killer (NK) cells, monocytes, neutrophils, Type 17 T helper cells were more common in cluster 2 (**Figure 5C**). It was confirmed in the validation set (**Figure 5D**) that cluster 2 might have stronger immune infiltration than cluster 1. Suggesting that it may have stronger immune cell infiltration than cluster 1. (ns, not significant, *P<0.05, **P<0.01, ***P<0.001).

### PPARα, NR1H4 (FXR) and SMAD3 expression in the BA and control groups

**Figure 6:**
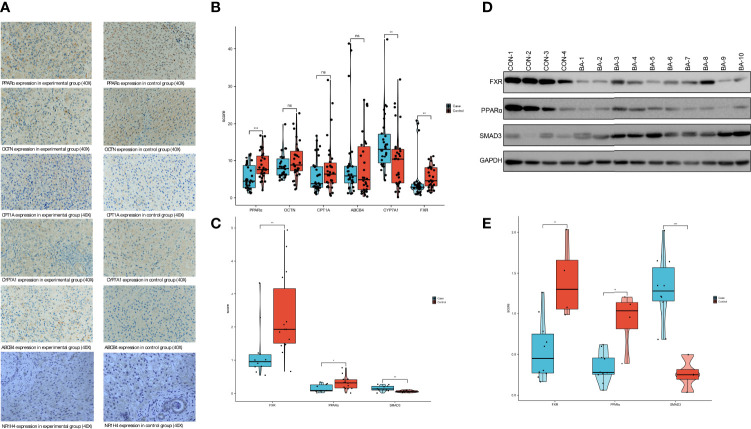
PPARα and NR1H4 (FXR) expression in the BA and control groups **(A)** IHC findings in the BA and control groups. **(B)** Semi-quantitative analysis of IHC in pathological sections using ImageJ software. (ns, not significant, *P<0.05, **P<0.01, ***P<0.001). **(C)** Verification of PPARα、FXR and SMAD3 expression in liver using RT-qPCR. The P values are labeled using asterisks (ns, no significance, *P < 0.05, **P < 0.01,***P < 0.001). **(D)** The expression of 3 mRNAs related protein in liver tissue detected by Westernblot. **(E)** The WB results were semi-quantitatively analyzed by ImageJ software.

Samples from the control group showed complete hepatic lobule structure was with hepatocellular cords lined up in an orderly manner. In the BA group, the extrahepatic bile duct was dilated and occluded to varying degrees, and the hepatic lobule and fibrous tissue around the bile duct were hyperplastic. Hepatocyte degeneration and necrosis were observed. The hepatic lobule structure and hepatic plate arrangement were disturbed, and intracellular cholestasis was noted (**Figure 6A**).The samples did not meet the requirements of normality testing (P<0.05), so differences were compared with Mann–Whitney U tests. Quantitative comparisons of IHC results are shown in **Figure 6B** and **Table 4**.

**Table 4:** Semi-quantitative ImageJ analysis of pathological sections.

	Case	Control	P
PPARα	5.409±3.39	8.741±3.756	<0.01
OCTN	8.467±3.356	10.17±4.952	>0.05
CPT1A	6.033±4.618	8.211±7.22	>0.05
CYP7A1	14.803±7.608	10.347±7.996	<0.01
MDR3	8.905±10.338	8.485±8.333	>0.05
NR1H4 (FXR)	4.323±5.32	5.495±2.887	<0.01

According to the results of PCR, we confirmed NR1H4 (FXR) expression in the BA group was 1.257±0.878 compared to 2.352±1.276 in the control group, which was statistically significant (P<0.01). PPARα expression levels were similar in BA and control groups (0.152±0.116 and 0.318±0.2, respectively; P<0.05). SMAD3 expression levels were significantly different between the BA and control groups (0.153±0.086 and 0.061±0.03, respectively; P<0.01) (**Figure 6C** and **Table 5**).

**Table 5:** PCR results(Wilcoxon rank sum test).

*Group*	*Group I [n]*	*Group II [n]*	*95% CI*	*P value*
*NR1H4(FXR)*	Case [10]	Control [14]	0.411 - 1.523	0.009
*PPARα*	Case [10]	Control [14]	0.033 - 0.299	0.026
*SMAD3*	Case [10]	Control [14]	-0.162 --0.034	0.004

CI, confidence interval.

The WB results showed that the mean levels of NR1H4 (FXR) expression in the BA and control groups were 0.555±0.376 and 1.407±0.482, respectively. The mean level of PPARα expression in the BA group was 0.339±0.185, compared to 0.914±0.366 in the control group. The mean levels of SMAD3 expression were 1.29±0.414 and 0.258±0.189 in the BA and control groups, respectively. The observed variables in each group were close to normal distribution (P>0.05) so t-tests were selected. The results showed that control group NR1H4 (FXR) expression was significantly higher than in the BA group (t=3.556, P<0.01). The same pattern was observed for PPARα expression (t=3.988, P<0.01). However, the expression level of SMAD3 was higher in the experiment(t=-4.708, P<0.01). Such results coincided with mRNA expression (**Figures 6D, E** and **Table 6**).

**Table 6:** Semi-quantitative WB results (T test).

*Group*	*Group I [n]*	*Group II [n]*	*95% CI*	*P value*
*NR1H4(FXR)*	Case [10]	Control [4]	0.33 - 1.375	0.004
*PPARα*	Case [10]	Control [4]	0.261- 0.888	0.002
*SMAD3*	Case [10]	Control [4]	-1.51 –0.554	0.001

CI, confidence interval.

## Discussion

Bioinformatics analysis revealed reduced PPARα and NR1H4 (FXR) mRNA levels in liver tissue from patients with BA. The TGFβ pathway is considered an important way to promote BA fibrosis (18–20). Activation of the TGFβ pathway is closely associated with low expression of PPARα and NR1H4, indicating that enhancing PPARα and NR1H4 levels may delay the progression of liver fibrosis. Furthermore, the PPI network results indicated that SMAD3 plays an important role in the aberrant activation of TGFβ signaling caused by low expression of PPARα and NR1H4. We found that SMAD3 levels significantly negatively correlated with PPARα and NR1H4 expression, indicating that their functions may have specific interferences or interactions. In animal experiments, the carnitine transporter OCTN2 can downregulate expression of TGFβ pathway genes in the intestinal epithelium of mice, and severe carnitine deficiency is associated with increased intestinal epithelial cell apoptosis, villous atrophy, intestinal inflammation and damage (21). The same mechanism might play a role in BA. We performed consensus clustering to classify BA patients in the GSE46960 dataset into two clusters: cluster 1 (low expression of SMAD3, high expression of PPARα and NR1H4) and cluster 2 (high expression of SMAD3, low expression of PPARα and NR1H4). The results indicated that cluster 2 had stronger immune cell infiltration than cluster 1, specifically for CD56dim NK cells, monocytes, and type 17 T helper cells. Dendritic cells are the most important antigen-presenting cells that initiate and maintain immune responses. The dendritic cell-Th17-macrophage axis was identified as a potential target for the treatment of BA (22). In another study, infection with human immunodeficiency virus (HIV) and hepatitis C virus (HCV) was closely associated with loss of CD56 (+), NK cells, and replacement expression of defective CD56 (-), CD16 (+), NK cells was observed with HIV infection. HCV infection impairs the overall NK cell response (23, 24). CD56 (-) was previously shown to be highly expressed in the liver of BA patients (25). Our study demonstrated higher CD56 (-)expression in cluster 2. Perhaps increased expression of CD56 (-) in BA and the subsequent immune response are also affected by several viral infections, causing a series of pathological changes. Monocyte infiltration plays an important role in the development of angiogenesis in experimental hepatopulmonary syndrome after common bile duct ligation, with increased levels of monocytes in the lung and liver that are accompanied by a decrease in the number of circulating monocytes (26). Whether due to viral infection-induced edema or other mechanisms, BA is the first trigger mechanism for attracting neutrophils. Subsequent activation of Kupffer and bile duct cells leads to the recruitment of other inflammatory cells, and this sequence of events is thought to be the result of pathological changes in BA rather than a causal mechanism (27). MCs migrate to the liver and are activated with cholestasis after liver injury in recent animal studies. Inhibition of MCs reduces the tube response and liver fibrosis, allowing the organ to heal in animal studies. The introduction of MC mimics cholestasis. Liver disorders, and MC-derived TGFβ may be therapeutic targets for chronic cholestasis liver disease (28). Studies have shown that TGFβ can effectively activate hepatic stellate cells, thereby promoting the production and secretion of extracellular matrix (ECM) proteins and causing liver fibrosis (29). In tumors, the TGFβ signaling pathway has multiple roles including regulation of the tumor microenvironment and tumor cell behavior. Inhibiting TGFβ signaling restores the ECM, regulates tumor vasculature, reverses epithelial-mesenchymal transition (EMT), damages cancer stem cells, and can enhance the response to chemotherapy (30, 31). Activation of the TGFβ signaling pathway promotes hepatocyte EMT and fibrosis progression in animal models (32). *In vitro*, EMT is induced by TGFβ in bile duct epithelial cells, causing BA fibrosis (33). The bile ducts innate immune response to dsRNA virus infection may induce EMT in bile duct epithelial cells by increasing tissue sensitivity to TGFβ (34). Our experiments showed reduced expression of PPARα and NR1H4 (FXR) mRNAs and proteins in BA liver tissue, while SMAD3 has the opposite trend. Upregulation of OCTN by PPARα activation can be seen as a means of supplying sufficient carnitine to cells (9). At the same time, it can enhance CYP7A1 activity and increase the secretion of bile acids to reduce their levels in the liver (14, 35). Therefore, impaired activation of PPARα signaling impairs carnitine utilization, reduces bile acid synthesis and secretion, affects NR1H4 (FXR) activation, and reduces the liver’s ability to suppress inflammation. In this setting, there is likely to be apoptosis and fibrosis, while low expression of PPARα and NR1H4TGFβ activates the SMAD3-mediated TGFβ pathway, leading to the progression of BA and cirrhosis. Overall, cluster 2 with high SMAD3 expression and low PPARα and NR1H4 expression had more identified mechanisms that promote disease progression. Presumably, interventions that enhance PPARα and NR1H4 levels may reduce the immune response of BA patients, thereby improving their prognosis.

## Conclusion

Our bioinformatics analysis and experimental validation results confirmed reduced PPARα and NR1H4 (FXR) mRNA and protein levels in BA. PPARα and NR1H4 can affect TGFβ signaling through SMAD3. In addition, SMAD3, PPARα and NR1H4 may affect the immune microenvironment of BA patients. The downregulation of PPARα and NR1H4 (FXR) signaling pathway may be closely related to biliary atresia.

## Data availability statement

The datasets presented in this study can be found in online repositories. The names of the repository/repositories and accession number(s) can be found below: https://www.ncbi.nlm.nih.gov/genbank/, GSE46960; https://www.ncbi.nlm.nih.gov/genbank/, GSE15235; https://www.ncbi.nlm.nih.gov/genbank/, GSE65359; https://www.ncbi.nlm.nih.gov/genbank/, GSE61276; https://www.ncbi.nlm.nih.gov/genbank/, GSE46960; https://www.ncbi.nlm.nih.gov/genbank/, GSE112790.

## Ethics statement

Written informed consent was obtained from the minor(s)’ legal guardian/next of kin for the publication of any potentially identifiable images or data included in this article.

## Author contributions

YM and LL contributed equally to this work. YM, LL and ZG conceived the idea for the article and performed data analysis, data interpretation, and manuscript preparation. LL, FF, WW, and JM performed the data acquisition, ZL, GT, LZ, KT, and YW contributed to the critical review of the intellectual content of this manuscript. 

## Acknowledgments

We thank our colleagues and hospital for their help with this study during the COVID-19 pandemic. Special thanks to Professor Lv Zhibao for supporting our article.

## Conflict of interest

The authors declare that the research was conducted in the absence of any commercial or financial relationships that could be construed as a potential conflict of interest.

The handling editor declared a shared parent affiliation with the authors at the time of the review.

## Publisher’s note

All claims expressed in this article are solely those of the authors and do not necessarily represent those of their affiliated organizations, or those of the publisher, the editors and the reviewers. Any product that may be evaluated in this article, or claim that may be made by its manufacturer, is not guaranteed or endorsed by the publisher.

## References

[1] Bezerra JA Wells RG Mack CL Karpen SJ Hoofnagle JH Doo E. Biliary atresia: Clinical and research challenges for the twenty-first century. Hepatol (Baltimore Md.) (2018) 68:1163–73. doi: 10.1002/hep.29905 PMC6167205 29604222

[2] Yang L Mizuochi T Shivakumar P Mourya R Luo Z Gutta S. Regulation of epithelial injury and bile duct obstruction by NLRP3, IL-1R1 in experimental biliary atresia. J Hepatol (2018) 69:1136–44. doi: 10.1016/j.jhep.2018.05.038 PMC6314850 29886157

[3] Lemoine C Melin-Aldana H Brandt K Mohammad S Superina R. The evolution of early liver biopsy findings in babies with jaundice may delay the diagnosis and treatment of biliary atresia. J Pediatr Surg (2020) 55:866–72. doi: 10.1016/j.jpedsurg.2020.01.027 32216969

[4] Nio M. Japanese Biliary atresia registry. Pediatr Surg Int (2017) 33:1319–25. doi: 10.1007/s00383-017-4160-x 29039049

[5] Taylor SA Venkat V Arnon R Gopalareddy VV Rosenthal P Erinjeri J. Improved outcomes for liver transplantation in patients with biliary atresia since pediatric end-stage liver disease implementation: Analysis of the society of pediatric liver transplantation registry. J Pediatr (2020) 219:89–97. doi: 10.1016/j.jpeds.2019.12.023 32005543

[6] Superina R. Biliary atresia and liver transplantation: results and thoughts for primary liver transplantation in select patients. Pediatr Surg Int (2017) 33:1297–304. doi: 10.1007/s00383-017-4174-4 29030698

[7] Gong Z Wu Y Zheng L Chen L Lv Z. Can free carnitine or bilirubin in blood be used in neonatal screening for biliary atresia? Eur J Pediatr Surg (2020) 30:459–64. doi: 10.1055/s-0039-1698764 31600802

[8] Gong Z Xu WJ Tian GL Zhang T Lv Z. Neonatal intrahepatic cholestasis caused by citrin deficiency differentiated from biliary atresia. Eur J Pediatr Surg (2016) 26:255–9. doi: 10.1055/s-0035-1551566 25988746

[9] Eder K Ringseis R. The role of peroxisome proliferator-activated receptor alpha in transcriptional regulation of novel organic cation transporters. Eur J Pharmacol (2010) 628:1–5. doi: 10.1016/j.ejphar.2009.11.042 19941851

[10] Al-Aqil FA Monte MJ Peleteiro-Vigil A Briz O Rosales R González R. Interaction of glucocorticoids with FXR/FGF19/FGF21-mediated ileum-liver crosstalk. Biochim Biophys Acta Mol Basis Dis (2018) 1864:2927–37. doi: 10.1016/j.bbadis.2018.06.003 29883717

[11] Zhou J Cui S He Q Guo Y Pan X Zhang P. SUMOylation inhibitors synergize with FXR agonists in combating liver fibrosis. Nat Commun (2020) 11:240. doi: 10.1038/s41467-019-14138-6 31932588 PMC6957516

[12] Xiao Y Yan W Zhou K Cao Y Cai W. Glucocorticoid treatment alters systemic bile acid homeostasis by regulating the biosynthesis and transport of bile salts. Dig Liver Dis (2016) 48:771–9. doi: 10.1016/j.dld.2016.03.022 27133208

[13] Ortiz-Perez A Donnelly B Temple H Tiao G Bansal R Mohanty SK. Innate immunity and pathogenesis of biliary atresia. Front Immunol (2020) 11:329. doi: 10.3389/fimmu.2020.00329 32161597 PMC7052372

[14] Kersten S Stienstra R. The role and regulation of the peroxisome proliferator activated receptor alpha in human liver. Biochimie (2017) 136:75–84. doi: 10.1016/j.biochi.2016.12.019 28077274

[15] Subramanian A Tamayo P Mootha VK Mukherjee S Ebert BL Gillette MA. Gene set enrichment analysis: A knowledge-based approach for interpreting genome-wide expression profiles. Proc Natl Acad Sci U.S.A. (2005) 102:15545–50. doi: 10.1073/pnas.0506580102 PMC1239896 16199517

[16] Wilkerson MD Hayes DN. ConsensusClusterPlus: a class discovery tool with confidence assessments and item tracking. Bioinformatics (2010) 26:1572–3. doi: 10.1093/bioinformatics/btq170 PMC2881355 20427518

[17] Newman AM Liu CL Green MR Gentles AJ Feng W Xu Y. Robust enumeration of cell subsets from tissue expression profiles. Nat Methods (2015) 12:453–7. doi: 10.1038/nmeth.3337 PMC4739640 25822800

[18] Mavila N James D Shivakumar P Nguyen MV Utley S Mak K. Expansion of prominin-1-expressing cells in association with fibrosis of biliary atresia. Hepatology (2014) 60:941–53. doi: 10.1002/hep.27203 PMC4146699 24798639

[19] Zagory JA Nguyen MV Dietz W Mavila N Haldeman A Grishin A. Toll-like receptor 3 mediates PROMININ-1 expressing cell expansion in biliary atresia *via* transforming growth factor-beta. J Pediatr Surg (2016) 51:917–22. doi: 10.1016/j.jpedsurg.2016.02.054 27059791

[20] Zhao D Luo Y Xia Y Zhang JJ Xia Q. MicroRNA-19b expression in human biliary atresia specimens and its role in BA-related fibrosis. Dig Dis Sci (2017) 62:689–98. doi: 10.1007/s10620-016-4411-z 28083843

[21] Sonne S Shekhawat PS Matern D Ganapathy V Ignatowicz L. Carnitine deficiency in OCTN2-/- newborn mice leads to a severe gut and immune phenotype with widespread atrophy, apoptosis and a pro-inflammatory response. PloS One (2012) 7:e47729. doi: 10.1371/journal.pone.0047729 23112839 PMC3480427

[22] Lages CS Simmons J Maddox A Jones K Karns R Sheridan R. The dendritic cell-T helper 17-macrophage axis controls cholangiocyte injury and disease progression in murine and human biliary atresia. Hepatology (2017) 65:174–88. doi: 10.1002/hep.28851 PMC5191928 27641439

[23] Fauci AS Mavilio D Kottilil S. NK cells in HIV infection: paradigm for protection or targets for ambush. Nat Rev Immunol (2005) 5:835–43. doi: 10.1038/nri1711 16239902

[24] Hong HS Eberhard JM Keudel P Bollmann BA Ahmad F Ballmaier M. Phenotypically and functionally distinct subsets contribute to the expansion of CD56-/CD16+ natural killer cells in HIV infection. Aids (2010) 24:1823–34. doi: 10.1097/QAD.0b013e32833b556f 20543659

[25] Okamura A Harada K Nio M Nakanuma Y. Participation of natural killer cells in the pathogenesis of bile duct lesions in biliary atresia. J Clin Pathol (2013) 66:99–108. doi: 10.1136/jclinpath-2012-201097 23162109

[26] Wu W Zhang J Yang W Hu B Fallon MB. Role of splenic reservoir monocytes in pulmonary vascular monocyte accumulation in experimental hepatopulmonary syndrome. J Gastroenterol Hepatol (2016) 31:1888–94. doi: 10.1111/jgh.13388 PMC5132097 27029414

[27] Changho S Ahmed AA. Neutrophils in biliary atresia. a study on their morphologic distribution and expression of CAP37. Pathol Res Pract (2010) 206:314–7. doi: 10.1016/j.prp.2010.02.001 20399025

[28] Kyritsi K Kennedy L Meadows V Hargrove L Demieville J Pham L. Mast cells induce ductular reaction mimicking liver injury in mice through mast cell-derived transforming growth factor beta 1 signaling. Hepatology (2021) 73:2397–410. doi: 10.1002/hep.31497 PMC7864988 32761972

[29] Liu Z Li C Kang N Malhi H Shah VH Maiers JL. Transforming growth factor β (TGFβ) cross-talk with the unfolded protein response is critical for hepatic stellate cell activation. J Biol Chem (2019) 294:3137–51. doi: 10.1074/jbc.RA118.005761 PMC6398135 30610118

[30] Chen J Ding ZY Li S Liu S Xiao C Li Z. Targeting transforming growth factor-β signaling for enhanced cancer chemotherapy. Theranostics (2021) 11:1345–63. doi: 10.7150/thno.51383 PMC7738904 33391538

[31] Ebbing EA Steins A Fessler E Stathi P Lesterhuis WJ Krishnadath KK. Esophageal adenocarcinoma cells and xenograft tumors exposed to erb-b2 receptor tyrosine kinase 2 and 3 inhibitors activate transforming growth factor beta signaling, which induces epithelial to mesenchymal transition. Gastroenterology (2017) 153:63–76.e14. doi: 10.1053/j.gastro.2017.03.004 28286209

[32] Weng T Yan D Shi D Zhu M Liu Y Wu Z. The MSP-RON pathway regulates liver fibrosis through transforming growth factor beta-dependent epithelial-mesenchymal transition. Liver Int (2021) 41:1956–68. doi: 10.1111/liv.14892 33786995

[33] Xiao Y Zhou Y Chen Y Zhou K Wen J Wang Y. The expression of epithelial-mesenchymal transition-related proteins in biliary epithelial cells is associated with liver fibrosis in biliary atresia. Pediatr Res (2015) 77:310–5. doi: 10.1038/pr.2014.181 25406900

[34] Harada K Sato Y Ikeda H Isse K Ozaki S Enomae M. Epithelial-mesenchymal transition induced by biliary innate immunity contributes to the sclerosing cholangiopathy of biliary atresia. J Pathol (2009) 217:654–64. doi: 10.1002/path.2488 19116990

[35] Zhang Y Lickteig AJ Csanaky IL Klaassen CD. Editor's highlight: Clofibrate decreases bile acids in livers of Male mice by increasing biliary bile acid excretion in a PPARα-dependent manner. Toxicol Sci (2017) 160:351–60. doi: 10.1093/toxsci/kfx191 PMC5837458 28973556

